# Osteoma in the upper cervical spine: A case report and comprehensive literature review

**DOI:** 10.1016/j.ijscr.2023.108924

**Published:** 2023-10-05

**Authors:** Seyed Ali Nabavizadeh, Mohammadhossein Khorraminejad-Shirazi, Dena Firouzabadi, Sara S. Nabavizadeh, Seyed Hamed Jafari, Amirreza Dehghanian

**Affiliations:** a Otolaryngology Research Center, Department of Otolaryngology, Shiraz University of Medical Sciences, Shiraz, Iran; b Department of Pathology, School of Medicine, Shiraz University of Medical Sciences, Shiraz, Iran; c Student Research Committee, Shiraz University of Medical Sciences, Shiraz, Iran; d Clinical Pharmacy Department, Shiraz School of Pharmacy, Shiraz University of Medical Sciences, Shiraz, Iran; e Shahid Faghihi Hospital, Clinical Pharmacy Department, Shiraz University of Medical Sciences, Shiraz, Iran; f Medical Imaging Research Center, Shiraz University of Medical Sciences, Shiraz, Iran; g Trauma Research Center, Shiraz University of Medical Sciences, Shiraz, Iran; h Molecular Pathology and Cytogenetics Division, Department of Pathology, Shiraz University of Medical Sciences, Shiraz, Iran

**Keywords:** Osteoma, Bone tumor, Laminectomy, Spine, Cervical canal stenosis

## Abstract

**Introduction and importance:**

Osteoma is a benign, and usually asymptomatic bone tumor normally found in the skull and facial bones, although it can occasionally occur in the long bones and spine.

**Case presentation:**

In this article, we present a 49-year-old male patient who experienced progressive neck pain accompanied by left-sided radicular pain symptoms. Clinical investigation using various imaging techniques confirmed a bone-forming lesion located within the C1 vertebrae region. Treatment involved performing hemilaminectomy of C1 along with resection for complete removal of this extradural bone lesion, ultimately achieving symptom relief. Histopathological examination of the resected specimen leads to the diagnosis of osteoma. Along with reporting this case, we conducted a comprehensive literature review of the previously reported spinal osteoma cases.

**Clinical discussion:**

Histopathological examination confirmed the diagnosis of osteoma. A comprehensive literature review was conducted, revealing 16 previously reported cases of spinal osteoma. Among these, only one case involved the C1 vertebra and presented similar neurological symptoms. The review underscores the rarity of spinal osteomas and the importance of surgical intervention for symptom relief.

**Conclusion:**

Spinal osteomas are rare but should be considered in the differential diagnosis of patients presenting with neck pain and radicular symptoms. Surgical removal of the lesion is often necessary for symptom relief, as highlighted by our case and supported by the literature review. This case adds to the limited body of evidence on spinal osteomas and emphasizes the importance of a multidisciplinary approach for optimal patient outcomes.

## Highlights

• Osteomas are benign bone lesions, mostly near sinuses, but infrequent in the upper cervical spine
• Surgical intervention often necessary for symptomatic upper cervical spine osteomas
• Case study: 49-year-old male with neck pain and left-sided radicular symptoms
• Successful relief through hemilaminectomy and lesion resection
• Rarity of upper cervical spine osteomas necessitates continuous reporting and analysis
• Personalized management is essential considering diverse treatment modalities

## 1 Background

Osteomas are benign osteogenic, slow-growing tumors located mainly near the paranasal sinuses and orbital region, and are typically localized lesions without systemic dissemination. [1]. Osteomas affecting the upper cervical spine are exceedingly uncommon [2,3]; sensory deficits and clinical presentation due to canal stenosis are its main complications that necessitate prompt clinical attention. While osteomas arising from the nasal sinuses or orbital cavity can lead to sinusitis or exophthalmia, cranial osteomas generally remain clinically inconspicuous [4,5]. Treatment approaches depend on the lesion's location, size, and patient symptoms, with surgical intervention typically reserved for cases exhibiting symptoms or functional impairment [6]. Surgical intervention serves as the primary management approach for upper cervical spine osteoma. However, the anatomical complexity of this region presents distinct challenges during surgical procedures [7].

Here, we present a case study involving a patient who experienced neck pain radiating to his left hand due to an osteoma in their C1 vertebra. Both complaints were successfully resolved without any complications through hemilaminectomy and resection of an extradural bone lesion. Moreover, we conducted a comprehensive literature review of the 14 reported cases of spinal osteomas in the literature, with only one located in the C1 vertebra. This highlights the rarity of our case and underscores the need for reporting and analyzing such unique occurrences to enhance our understanding of osteomas in the upper cervical spine.

This work has been reported in line with the SCARE criteria to ensure standardized and high-quality reporting [8].

## 2 Case presentation

A 49-year-old man was referred to the neurosurgery department in Chamran Hospital, Fars, Shiraz, Iran, complaining of progressive neck pain and left-sided radicular pain persisting for a period of 6 months. Additionally, the patient experienced hypoesthesia and paresthesia in the left hand approximately two weeks before seeking medical attention. The patient denied any history of trauma prior to the onset of symptoms. Notably, the pain did not extend to the lower back or hips, and no numbness in the lower extremities was reported. Past medical and drug history was unremarkable, as was the patient's social history. The patient also reported no congenital abnormalities in the upper cervical spine. The patient exhibited normal cranial nerve function, muscle power, and gait.

**Fig. 1:**
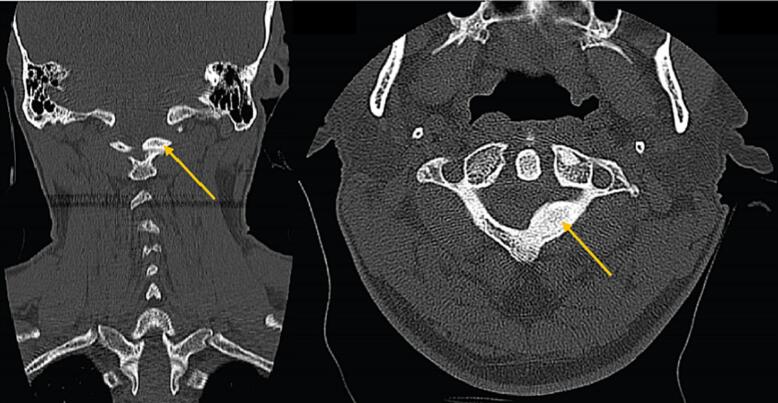
preoperative cervical computed tomography (CT) scan revealed the presence of a bony lesion on the left pedicle of the C1 vertebra that extends into the spinal canal.

Computed tomography (CT) scan displayed a well-delineated ovoid bony mass on the left side of C1 pedicle that mildly extended into the spinal canal, with cortical density on bone window without evidence of significant cord compression (Fig. 1). Magnetic resonance imaging (MRI) of the cervical spine indicated a mild deformity in the C1 left pedicle, resulting in thecal sac compression and mild canal stenosis, however, no atlanto-axial dislocation was observed. The evaluation determined that the sagittal diameter of the spinal canal at the C1 level was within the acceptable range of measurements. Based on the assessment, the impression was C1 benign bone forming tumor, leading to the patient undergoing an operation. During the procedure, a hemilaminectomy of C1 and total resection of the extradural bone lesion were performed.

**Fig. 2:**
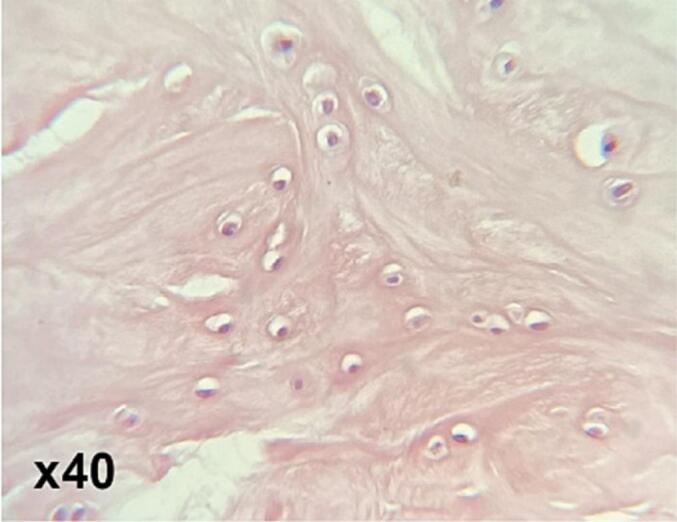
Histopathological analysis revealed that the lesion consisted of uniformly dense and compact cortical-like mature bone, without any cellular atypia consistent with the diagnosis of osteoma.

Histopathological examination of the resected specimens revealed fragments of mature cortical type bone architecture with areas of endochondral ossification, showing no cellular atypia or necrosis. The diagnosis of the specimens and extradural bone lesion was suggestive of osteoma (Fig. 2). The patient's post-operative course has been uneventful, with relief from symptoms and disappearance of radiating pain. Also, a postoperative CT scan confirmed the complete removal of the osteoma.

## 3 Discussion and conclusion

In this article, a case of osteoma in the C1 region is presented with upper cervical canal stenosis. Hemilaminectomy of C1 and complete resection of the extradural bone lesion was performed. Accurate diagnosis of osteomas in this anatomical region poses significant challenges as they have radiologic and clinical overlap with other medical conditions, making them difficult to detect [6]. Kanaya et al. reported a C1 osteoma while conventional CT and MRI scans in the neutral position did not reveal an obvious cause, a rotational myelo-CT scan showed a bony lesion leading to the diagnosis [2]. However, challenges in utilizing this technique among patients experiencing intense radiating pain during prolonged positioning limit its applicability in such cases [2]. This highlights the diagnostic challenges associated with osteomas of the cervical region. More to the point, other clinical entities like a unilateral atlantoaxial (C1-C2) pseudoarticulation and osteochondroma can mimic cervical osteoma making the radiologic diagnosis difficult with conventional modalities like CT scan and MRI [7,9]. Of note, obtaining adequate tissue sampling and complete resection is critical for confirming the diagnosis when clinical suspicion for an osteoma exists., A.

We conducted a literature review of all cases of spinal osteoma, listed in Table 1 [2,3,6,7,[10], [11], [12], [13], [14], [15]]. These cases demonstrated similarities in terms of neurological symptoms and the necessity for surgery, resulting in symptom improvement postoperatively. However, there were variations in tumor location; among these cases, in eight of them the tumor was located in the cervical spine [2,3,[10], [11], [12], [13]], and in only two of the cases including the present case, the tumor was located in C1 vertebra [2]. The location of the rest of the osteomas presented varies between the suboccipital bone [7], thoracic [[14], [15], [16]], lumbar [6,12], and sacrum [12]. The rarity of osteomas in the C1 region was evident from the limited number of reported cases in the literature. Furthermore, a study by Peyser et al. reported five patients who underwent subtotal resection of the lesion, indicating that relief of symptoms cannot be guaranteed, and indicated that total resection should be considered as the preferred approach [12], although Wang et al. in 2006 presented a case with C2 lamina osteoma that underwent laminectomy of C2 and C3, partial removal of the tumor led to significant improvement in symptoms [3]. This variety of cases emphasizes the importance of personalized treatment strategies; partial tumor removal [3,12], focused decompression [7], and total tumor removal [2,3,6,7,[10], [11], [12], [13],^16^] were employed in some cases, suggesting the effectiveness of diverse surgical methods in managing cervical osteomas.

**Table 1:** Reported cases of spinal osteoma.

Author/year	Age/sex	Tumor location	Sign and Symptoms	Surgery	Outcome
Present case	49/M	C1 vertebral pedicle	neck pain and left-sided radicular pain	Hemilaminectomy of C1 and total resection	Relief of symptoms, confirmed by postoperative imaging
Shigekawa et al.,2022 [16]	45/M	Dorsal T6 body	Numbness in left chest and lower extremities, dysesthesia in Th6–8 region, hyperreflexia in lower limbs	Laminoplasty of T6, total resection	Complete tumor removal, uneventful recovery with no sequelae
Son al., 2022 [7]	49/F	Left suboccipital bone	Pain in left side of face, jaw, eye, neck, shoulder, arm, and leg	Decompression of greater occipital nerve	Significant improvement, 70 % reduction in pain, resumed daily activities
Forlizzi et al., 2019 [14]	47/M	T3–4 facet joints	Lower-extremity radiculopathy, “electric shock” sensations in right posterior leg down calf, sexual dysfunction	laminectomy with three-level posterior spinal fusion from T2-T4	Immediate symptom relief, returned to work by 6-month follow-up, no subjective numbness or pain medication usage
Munakomi et al., 2019 [15]	55/M	Epidural at T6	Progressive weakness of bilateral lower limbs, altered sensation up to the T4 dermatome, hypertonia, clonus | Thoracic laminectomy with drilling of solid bony lesion	Thoracic laminectomy with drilling of solid bony lesion	No immediate clinical improvement, advised for continuous physiotherapy
Kanaya et al., 2016 [2]	43/F	Posterior C1 vertebra	Numbness, pain in right hand, aggravated by neck rotation	Removal of bony lesion	Relief of symptoms, confirmed by postoperative imaging
Aniba et al., 2011 [10]	45/F	C2 vertebral body	Paresthesia in all four limbs, walking fatigue resembling claudication	Complete resection of exostosis, C2 laminectomy	Significant improvement in walking, disappearance of osteoma
Wang et al., 2006 [3]	56/M	C2–3 vertebral body	Paresis, weakness, limited neck rotation, hypoesthesia	Laminectomy of C2 and C3, partial removal	Significant improvement in symptoms, increased muscle strength, improved daily activities
	49/M	Left C2 lamina	Paresis, weakness, hypoesthesia, positive reflexes, ankle clonus	Laminectomy of C3-C4 and part of C2	Significant improvement in symptoms, increased muscle strength, improved walking
Kobayashi et al.,2006 [6]	57/M	L5 articular process	Chronic low back pain, sciatica	Total resection (L5-S1 TLIF)	Resolved
Rengachary et al.,1998 [11]	34/M	C6 vertebral pedicle	Weakness, numbness, tingling in the right arm, neck pain	Not specified	Not specified
Laus et al.,1996 [13]	53/M	C2–3 transverse process	Dysphagia	Unknown	Resolved
Peyser et al.,1996 [12]	44/F	L4 body	Low back pain	Total resection (L4–5 PLIF)	Resolved
	64/F	C4–6 body	Chronic neck pain, weakness, paresthesia	subtotal resection (C2–6 PF)	Resolved, but pain recurred
	68/F	Ala of sacrum	Chronic low back pain	total resection (L4-S1 PLF)	Resolved, but pain recurred
	43/F	S2 body	Low back pain	Total resection	No change
	63/F	L5 body	Chronic low back pain, sciatica	Total resection (L4-S1 ALIF)	Resolved

Abbreviations: ALIF: (anterior lumbar interbody fusion), TLIF: (transforaminal lumbar interbody fusion), PF: (posterior spinal fusion), PLF: (posterolateral lumbar fusion).

In conclusion, our case report and comprehensive literature review enhance the understanding of osteomas in the upper cervical spine. By presenting a rare case of osteoma in the atlas and analyzing existing studies, we contribute to the body of knowledge surrounding the accurate diagnosis and surgical management of these lesions.

## Abbreviations

C1: First cervical vertebra
C2: Second cervical vertebra
C3: third cervical vertebra
CT: Computed Tomography
MRI: Magnetic Resonance Imaging

## Ethics approval and consent to participate

The present study was approved by the Medical Ethics Committee of XXX University of Medical Sciences. The purpose of this report was completely explained to the patient and written inform consent was obtained from the patient.

## Consent for publication

Written informed consent was obtained from the patient for publication of this case report and accompanying images. A copy of the written consent is available for review by the Editor-in-Chief of this journal on request.

## Data avaibility

Data of the patient can be requested from authors. Please write to the corresponding author if you are interested in such data.

## Funding

No source of funding.

## CRediT authorship contribution statement

SAN and SSN drafted the manuscript.MK collected the data and revised the manuscript. SHJ collected and reported the imaging data. DF proofread the manuscript and did the English language editing of the manuscript. AD revised the final version of the manuscript and supervised the study. All authors read and approved the final version of the manuscript.

## Guarantor

Seyed ali nabavi zadeh accept full responsibility for the work and/or the conduct of the study,

had access to the data, and controlled the decision to publish.

## Declaration of competing interest

The authors declare that they have no competing interests.
